# Population Growth of Three Stored Product Beetle Species on *Alphitobius diaperinus* (Coleoptera: Tenebrionidae) Meals

**DOI:** 10.1093/jee/toad025

**Published:** 2023-03-08

**Authors:** M Rigopoulou, C I Rumbos, C G Athanassiou

**Affiliations:** Laboratory of Entomology and Agricultural Zoology, Department of Agriculture, Crop Production and Rural Environment, University of Thessaly, Phytokou Street, 38446, Volos, Magnesia, Greece; Laboratory of Entomology and Agricultural Zoology, Department of Agriculture, Crop Production and Rural Environment, University of Thessaly, Phytokou Street, 38446, Volos, Magnesia, Greece; Laboratory of Entomology and Agricultural Zoology, Department of Agriculture, Crop Production and Rural Environment, University of Thessaly, Phytokou Street, 38446, Volos, Magnesia, Greece

**Keywords:** edible insect, insect infestation, insect meal, lesser mealworm, population growth

## Abstract

The lesser mealworm, *Alphitobius diaperinus* (Panzer) (Coleoptera: Tenebrionidae), is an important pest of stored products and at the same time a species with great potential as food and feed that has recently attracted increasing interest as nutrient source. Future predictions show a massive increase of insect meals’ production in the near future, therefore, as expected in the case of other durable stored products, insect meals may get infested by insects during their storage. In this context and as a continuance of our previous study on the susceptibility of meal of the yellow mealworm, *Tenebrio molitor* L. (Coleoptera: Tenebrionidae), to storage insect infestations, the objective of this study was to test the susceptibility of *A. diaperinus* meals to infestations of three stored products pests, namely *A. diaperinus* itself, *T. molitor*, and the red flour beetle, *Tribolium castaneum* (Herbst) (Coleoptera: Tenebrionidae). The population growth of the three species was evaluated on pure *A. diaperinus* meal, as well as on *A. diaperinus* meal-based substrates with different percentages of wheat bran (0, 25, 50, 90, and 100% bran). Our results showed that all three insect species tested were able to grow and develop on the *A. diaperinus* meal-based substrates evaluated, giving in a short time high population densities. This study confirms again our initial theory for insect infestations during storage of insect-based products.

The lesser mealworm, *Alphitobius diaperinus* (Panzer) (Coleoptera: Tenebrionidae), is an important pest of stored products that has been found infesting dozens of agri-industrial commodities (Hagstrum and Subramanyam 2009, Hagstrum et al. 2013). Moreover, this species can be a carrier of certain diseases that can seriously endanger human and animal health, while it is considered as one of the most serious pests in poultry farms (Rumbos et al. 2019). Despite being a noxious pest, *A. diaperinus* is among the first list of species that had been authorized for aquafeed production in 2017 in the European Union (EU) [Commission Regulation 2017/893 (EC 2017)], an authorization that has been recently expanded to poultry and swine feed [Commission Regulation 2021/1372 (EC 2021)], but also to human consumption (Turck et al. 2022). Currently, most commercial insect producing units in the EU are focused on the production of the black soldier fly, *Hermetia illucens* (L.) (Diptera: Stratiomyidae), and the yellow mealworm, *Tenebrio molitor* L. (Coleoptera: Tenebrionidae) (Rumbos and Athanassiou 2021), but there has been recently an increasing research interest for the mass rearing of *A. diaperinus*, especially on the utilization of different durable agricultural commodities and their by-products (Rumbos et al. 2021, Gourgouta et al. 2022a).

The projections of the increase in global edible insects market in terms of volume indicate that until 2030 the quantities that will be produced are expected to exceed 3 million tonnes (Meticulous Market Research 2022). Hence, the increase in production will concomitantly require an analogous increase in the need for storage and processing facilities, in terms of both storage capacity and duration. As in the case of other durable stored products, these insect-based ‘products’ are expected to be infested by the typical stored product insects that are perfectly adopted to develop at the postharvest stages of agricultural commodities. However, studies on the susceptibility of insect meals and related insect-based products to insect infestations during storage are limited. A first attempt to widely quantify this hypothesis was that of Rumbos et al. (2020), who examined the vulnerability of *T. molitor* meals to infestations caused by different stored product beetle species, including *T. molitor* itself. The rationale behind this study was simple: if there is an infestation in insect rearing units, then control can be a very complicated procedure, and any control methods should be extremely selective (Rumbos et al. 2020). For instance, there are certain compounds, such as diatomaceous earths, that are very effective for the control of many insect pests, but not for the control of *T. molitor* larvae (Mewis and Ulrichs 2001, Gourgouta et al. 2022b). Additional studies suggest that the use of selective parasitoids could provide a solution (Deruytter et al. 2021). However, the application of these techniques literarily means that diatomaceous earths or parasitoids should be added into the facility and/or the commodity, which is not always desirable. Newer data underline the importance of this phenomenon that is becoming an increasing problem at the industrial level (Deruytter et al. 2021).

As a continuance of the Rumbos et al. (2020) study for the insect infestations in *T. molitor* meals, we considered to expand this idea with the current work to *A. diaperinus* meals, testing the population growth of three insect species, which were *A. diaperinus* itself, *T. molitor* and the red flour beetle, *Tribolium castaneum* (Herbst) (Coleoptera: Tenebrionidae), which is a major pest of stored products with global distribution (Campbell et al. 2022).

## Materials and Methods

### Insect Rearing

All insect species used in the bioassay were reared at the Laboratory of Entomology and Agricultural Zoology, Department of Agriculture, Crop Production and Rural Environment, University of Thessaly. Specifically, *T. castaneum* was reared on flour, *T. molitor* was reared using wheat bran, while for *A. diaperinus* a mixture of wheat bran and egg layer hens pellets (3:1) was used. *T. molitor* and *A. diaperinus* were weekly supplied with fresh potatoes and apples, respectively, as a moisture source. *T. castaneum* and *T. molitor* were kept at 26°C, 55% relative humidity (RH), whereas *A. diaperinus* was kept at 30°C and 55% RH. All insect species were kept at continuous darkness. The adults used in the tests were <1 month-old.

### Insect Meal Preparation

For the *A. diaperinus* meal preparation late-stage larvae were used, after harvesting them by separating them from the feeding substrate with sieving. Larvae were frozen at −20°C, chopped using a stainless-steel mill (Thermomix TM31-1, Vorwerk Elektrowerke GmbH & Co. KG, Wuppertal, Germany), dried for 72 h at 60°C, and finally sieved with an 1-mm opening sieve. The insect meal produced was stored at −20°C until the initiation of the bioassays.

### Experimental Design

The population growth of *T. castaneum*, *T. molitor*, and *A. diaperinus* was evaluated in a series of laboratory bioassays, in which pure *A. diaperinus* meal was used, as well as mixtures of it with wheat bran in different percentages. Specifically, five substrates based on insect meal were evaluated: 100% *A. diaperinus* meal (0% wheat bran), a mixture of 75% *A. diaperinus* meal and 25% wheat bran, a mixture of 50% *A. diaperinus* meal and 50% wheat bran, a mixture of 10% *A. diaperinus* meal and 90% wheat bran and 100% wheat bran (0% *A. diaperinus* meal). Wheat bran was selected as substrate ingredient, as it is a major feed commodity, commonly used for animal livestock feeding (Fuller 2004). All substrates used in the bioassays were clean and free of insect infestations. The whole experiment procedure was conducted in cylindrical plastic vials (Rotilabo-sample tins with snap-on lid, 7.5 cm in diameter, 8.5 cm in height, Carl Roth Gmbh & Co. Kg, Karlsruhe, Germany), on which an opening (6.0 cm diameter) in the lid was created and covered with muslin gauze in order to ensure proper aeration of the vial. Polytetrafuoroethylene preparation (Fluon, 60 wt% dispersion in water, Sigma-Aldrich Chemie GmbH, Steinheim, Germany) was applied on the upper and inner part of the vials to prevent insects from escaping the vials. Ten grams of each of the mentioned substrates were placed in the vials, using different vials for each substrate. Finally, 20 adults of each of the three insect species tested in the bioassays (i.e., *T. castaneum, T. molitor*, and *A. diaperinus*) were placed into the vials, using different vials for each species. All vials were then placed at the proper conditions for each species mentioned above and were kept there for 65 d. In the case of *T. molitor* and *A. diaperinus*, a slice of potato and carrot, respectively, was placed inside the vials twice a week to cover the insect moisture needs. After 65 d the vials were opened for the evaluation of the progeny production and the produced adults (alive or dead), larvae, and pupae were counted. As a direct measure of population growth, for all species, the instantaneous rate of increase was calculated using the following equation: r_i_ = ln(N_f_/N_o_)/ΔT, where N_f_ was the final number of individuals, N_o_ was the initial number of individuals, and ΔT was the change in time, i.e., the duration of the experiment. Positive values of r_i_ suggest a growing population, r_i_ = 0 shows a stable population, and negative r_i_ values specify a population in decline (Stark and Banks 2003). There were three replicates for each treatment (tree vials replicates) with the whole procedure been repeated three times (three series of vials) by preparing new vials each time (3 × 3 = 9 vials for each treatment).

### Statistical Analysis

At first, the data were checked for normality using Shapiro–Wilk’s test (Zar 1999). For all species tested, data for the different life stages that were evaluated, as well as the instantaneous rates of increase, were tested for normality and found non-normally distributed. Therefore, data were compared with the Kruskal–Wallis H-test followed by multiple pairwise Mann–Whitney *U* tests using Bonferroni correction (Zar 1999). For all species, the total number of individuals at the end of the bioassay was modeled as a function of the percentage of the insect meal in the substrate, applying regression analysis, and a curve-fitting approach. Suitability of models and fitting curves was determined by examination of the correlation coefficient. All analyses were conducted using the IBM SPSS Statistics software, Version 25 (IBM Corporation, Armonk, NY).

## Results

### Tribolium castaneum

**Fig. 1.**
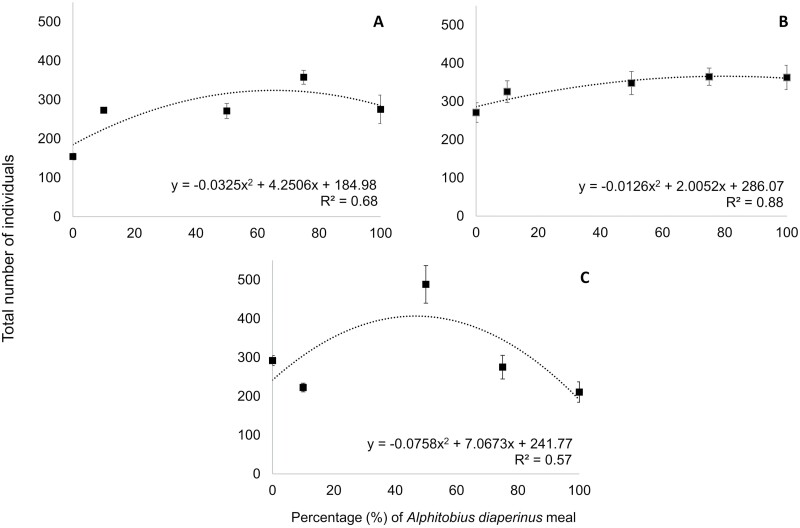
Total number of individuals of *Tribolium castaneum* (A), *Alphitobius diaperinus* (B), and *Tenebrio molitor* (C) after 65 d on substrates based on *A. diaperinus* meal and wheat bran at different percentages (0, 10, 50, 75, and 100% *A. diaperinus* meal). Equations are curve-fit equations that best fit to the raw data (*n* = 9). The R2 of the equation is also given for each curve.

The results showed that *T. castaneum* could grow and develop on pure *A. diaperinus* meal, as a high number of larvae and pupae (71 larvae and 76 pupae in total per vial) was counted in this treatment by the end of the bioassay (Table 1). More specifically, in this treatment an almost 14-fold increase in *T. castaneum* population was recorded, as the initially inserted 20 adults produced in 65 d a total population of 275 individuals per vial, giving an instantaneous rate of increase of 0.039. A high progeny production was also recorded on the rest of the substrates evaluated, i.e., on the mixtures of *A. diaperinus* meal with wheat bran in different percentages (0 to 75% *A. diaperinus* meal), as the total number of the produced individuals ranged from 154 to 358 at the termination of the bioassay, corresponding to instantaneous rates of increase ranging between 0.031 and 0.044. Significantly higher instantaneous rates of increase were recorded on the mixtures of the insect meal with wheat bran, i.e., on the substrates with 10, 50, and 75% *A. diaperinus* meal, than on wheat bran alone. The population growth of *T. castaneum*, expressed as total number of individuals at the end of the bioassay, as a function of the percentage of *A. diaperinus* meal in the substrates was best described by a quadratic equation (*R*^2^ = 0.68, Fig. 1A).

**Table 1.** Population growth [mean number of individuals (±SE) per vial] and instantaneous rate of increase of *Tribolium castaneum* after 65 d on substrates based on *Alphitobius diaperinus* meal and wheat bran at different percentages (0,10, 50, 75, and 100% *A. diaperinus* meal) (*n* = 9).

Substrates	Adults (dead)	Adults (alive)	Larvae	Pupae	Total	Instantaneous rate of increase
100% *A. diaperinus* meal (0% wheat bran)	6.0 ± 1.7BC	122.4 ± 18.5BC	70.7 ± 8.3AB	76.2 ± 16.1AB	275.3 ± 36.5 AB	0.0393 ± 0.0020 AB
75% *A. diaperinus* meal (25% wheat bran)	13.9 ± 2.0ABC	167.0 ± 10.2AB	94.7 ± 7.0A	82.0 ± 7.1A	357.6 ± 18.0 A	0.0443 ± 0.0008 A
50% *A. diaperinus* meal (50% wheat bran)	23.6 ± 2.2A	183.3 ± 8.6AB	42.8 ± 8.4B	21.6 ± 4.4C	271.2 ± 19.4 AB	0.0399 ± 0.0011 A
10% *A. diaperinus* meal (90% wheat bran)	16.3 ± 1.8AB	199.1 ± 6.3A	37.9 ± 3.8A	19.9 ± 4.0C	273.2 ± 7.8 AB	0.0403 ± 0.0004 A
0% *A. diaperinus* meal (100% wheat bran)	3.3 ± 0.4C	83.2 ± 5.0C	36.3 ± 3.2B	31.4 ± 6.7BC	154.3 ± 8.5 B	0.0311 ± 0.0009 B
χ^2^	30.9	27.5	23.9	27.7	26.8	26.7
*P*	<0.001	<0.001	<0.001	<0.001	<0.001	<0.001

Within each column, means followed by the same uppercase letter are not significantly different [in all cases, df = 4, 44; Mann–Whitney *U* test at 0.05]. Where no letters exist, no significant differences were noted.

### Alphitobius diaperinus

Similarly to *T. castaneum*, *A. diaperinus* was able to grow and develop on 100% *A. diaperinus* meal. At the end of the trial, a high number of larvae was recorded on this substrate (342 larvae per vial) (Table 2). Additionally, the total number of individuals by the end of the bioassay on this substrate reached the 363 individuals per vial, which means a population growth by 18 times, with an instantaneous rate of increase of 0.044. Moreover, a high progeny production was also recorded on the mixtures of the *A. diaperinus* meal with wheat bran, with a total number of individuals ranging from 271 to 365 at the termination of the bioassay, corresponding to instantaneous rates of increase between 0.039 and 0.044. The total number of individuals at the end of the experiment on 100% insect meal, as well as on the mixtures of *A. diaperinus* meal with wheat bran, was higher compared to wheat bran, however differences were not significant (Table 2). A quadratic equation best fit the curve describing the correlation between the percentage of *A. diaperinus* meal in the substrates with the total number of individuals at the end of the trial (*R*^2^ = 0.88, Fig. 1B).

**Table 2.** Population growth [mean number of individuals (±SE) per vial] and instantaneous rate of increase of *Alphitobius diaperinus* after 65 d on substrates based on *A. diaperinus* meal and wheat bran at different percentages (0,10, 50, 75, and 100% *A. diaperinus* meal) (*n* = 9).

Substrates	Adults (dead)	Adults (alive)	Larvae	Pupae	Total	Instantaneous rate of increase
100% *A. diaperinus* meal (0% wheat bran)	1.0 ± 0.3	19.2 ± 0.3	342.0 ± 32.0	0.6 ± 0.3	362.8 ± 31.7	0.0442 ± 0.0013
75% *A. diaperinus* meal (25% wheat bran)	0.9 ± 0.3	19.3 ± 0.4	339.2 ± 24.1	5.1 ± 2.8	364.6 ± 22.6	0.0444 ± 0.0010
50% *A. diaperinus* meal (50% wheat bran)	1.1 ± 0.4	18.9 ± 0.4	323.2 ± 32.2	5.0 ± 2.3	348.2 ± 30.5	0.0436 ± 0.0014
10% *A. diaperinus* meal (90% wheat bran)	1.4 ± 0.3	18.8 ± 0.3	302.3 ± 28.8	3.0 ± 1.5	325.6 ± 28.5	0.0426 ± 0.0015
0% *A. diaperinus* meal (100% wheat bran)	1.0 ± 0.3	18.9 ± 0.3	249.2 ± 26.5	1.9 ± 1.0	271.0 ± 25.7	0.0394 ± 0.0017
χ^2^	1.8	2.4	6.1	2.9	6.4	6.7
*P*	0.781	0.663	0.195	0.581	0.170	0.154

Within each column, means followed by the same lowercase letter are not significantly different [in all cases, df = 4, 44; Mann–Whitney *U* test at 0.05]. Where no letters exist, no significant differences were noted.

### Tenebrio molitor

There were significant differences in the population growth of *T. molitor* among the substrates tested for all life stages examined, with the exception of dead adults (Table 3). Numerous larvae were counted on 100% *A. diaperinus* meal (194 larvae per vial) showing the ability of *T. molitor* to grow and develop on this substrate. However, the total number of individuals was lower on this substrate at the termination of the trial than on the rest of the substrates tested, differences though were significant only with the 50% *A. diaperinus* meal. For the latter substrate, at least 24-fold increase of the *T. molitor* population was recorded at the termination of the bioassay. As far as the instantaneous rates of increase of all substrates are concerned, those ranged between 0.035 and 0.048, with the value of the substrate with 50% insect meal being the highest one (0.048). The population growth of *T. molitor*, as a function of the *A. diaperinus* meal percentage in the tested substrates, was best described by a quadratic equation (*R* = 0.57, Fig. 1C).

**Table 3.** Population growth [mean number of individuals (±SE) per vial] and instantaneous rate of increase of *Tenebrio molitor* after 65 d on substrates based on *Alphitobius diaperinus* meal and wheat bran at different percentages (0,10, 50, 75, and 100% *A. diaperinus* meal) (*n* = 9).

Substrates	Adults (dead)	Adults (alive)	Larvae	Pupae	Total	Instantaneous rate of increase
100% *A. diaperinus* meal (0% wheat bran)	5.0 ± 1.3	12.8 ± 1.9A	193.7 ± 26.6B	4.4 ± 0.7B	210.9 ± 26.1B	0.0411 ± 0.0007AB
75% *A. diaperinus* meal (25% wheat bran)	6.6 ± 1.2	12.3 ± 1.4A	257.7 ± 30.9AB	6.6 ± 0.5AB	275.1 ± 30.6AB	0.0369 ± 0.0008B
50% *A. diaperinus* meal (50% wheat bran)	6.1 ± 0.5	3.4 ± 1.2C	478.8 ± 49.3A	9.4 ± 0.3A	488.3 ± 48.2A	0.0485 ± 0.0016A
10% *A. diaperinus* meal (75% wheat bran)	2.8 ± 0.5	11.9 ± 0.8AB	208.3 ± 11.0B	6.2 ± 0.1B	222.7 ± 11.2B	0.0395 ± 0.0019AB
0% *A. diaperinus* meal (100% wheat bran)	4.3 ± 0.9	4.9 ± 1.3BC	282.9 ± 13.1AB	4.8 ± 0.2B	292.1 ± 13.0AB	0.0348 ± 0.0027B
χ^2^	8.2	23.8	23.4	30.5	23.1	23.1
*P*	0.085	<0.001	<0.001	<0.001	<0.001	<0.001

Within each column, means followed by the same lowercase letter are not significantly different [in all cases, df = 4, 44; Mann–Whitney *U* test at 0.05]. Where no letters exist, no significant differences were noted.

## Discussion

The current study is a continuance of the previous work by Rumbos et al. (2020), where the authors illustrated that there are increased chances for *T. molitor* to be fed on *T. molitor* meal, along with *T. confusum*. Although the chances of ‘cross-infestation’ of *T. molitor* meal by *T. molitor* ‘escapees’ from the production line can be reduced, and, apparently, may not be highly undesirable for the final product, the occurrence of the species of the genus *Tribolium* can drastically affect the quality of *T. molitor* meal. The infestation by *T. castaneum*, apart from the qualitative degradations and the quantitative losses, can be related with the transmission of nonfood borne pathogens that may endanger human and animal health, inducing antibiotic resistance to the digestive system (Channaiah et al. 2010, Hubert et al. 2018, Parlapani et al. 2020). Indicatively, in a surveillance of different stored product beetle populations originated from various geographical zones, Parlapani et al. (2020) found enterococci in the vast majority of the populations, which some exhibiting increased resistance to some of the most commonly used antibiotics. At the same time, several stored product insect species, including *Tribolium* spp., can produce high amounts of quinones that have been considered as carcinogenic (Hubert et al. 2018, Campbell et al. 2022). In this context, the presence of stored product insects in the final ‘commodity’, i.e., the insect meals, may be related with serious detrimental effects, apart from the infestation per se. Our data clearly illustrate that all three species tested here can develop with ease in *A. diaperinus* meal.

From a commercial point of view, these infestations could be unavoidable, as it happens in the case of storage and processing facilities, like silos, warehouses, flour mills etc. Nevertheless, in these types of facilities the infestations can be controlled with the use of various chemicals, such as fumigants and contact insecticides, which apparently can be repeated whenever is needed (Athanassiou and Arthur 2018). In contrast, almost all of these methods cannot be applied in the case of insect-based meal facilities, as they will endanger the survival and affect the performance of the farmed insects. In a recent review paper, Deruytter et al. (2021) thoroughly discussed the growing phenomenon of insect infestations in insect producing facilities, caused particularly by Pyralid moths. In that work, the authors suggested that there might be some nonchemical control techniques that are species-specific, such as mating disruption, which can apparently control moth populations, but not Coleoptera, Diptera, or Orthoptera (Deruytter et al. 2021). However, there are no commercially available mating disruption formulations for the species tested here. The current work is based on the predictions that the insect meal production will be drastically increased in the very near future (Meticulous Market Research 2022), which will eventually require increased storage needs in terms of both the quantities that are to be stored and the interval that these quantities have to be stored before use. In this context, increased quantities may result in increased infestations by stored product insect species.

The progeny production data indicate that, although all three species tested here can develop in *A. diaperinus* meal, the increase of the percentage of insect meal in the diet over a point resulted is some cases in a reduction in the progeny production, along with a developmental delay. In the Rumbos et al. (2020) paper, there was a delay in the development of *T. molitor* larvae when they were fed exclusively with *T. molitor* meal, but the increase of the percentage of wheat bran to 25% accelerated pupal formation and adult emergence, while a further increase in the percentage of bran increased progeny production further. Our data show that, for *A. diaperinus* adults, mortality was not affected by the insect meal containment. Moreover, the number of emerged *A. diaperinus* individuals was increased with the increase of the insect meal containment in the diet; in fact, when the diet contained 100% wheat bran, the number of *A. diaperinus* individuals was lower than that on the other treatments, suggesting that the presence of even 10% of *A. diaperinus* meal is beneficial for the development of this species. *A. diaperinus* is considered as a ‘dirty feeder’ and can be developed on dead insects (Rumbos et al. 2019). As such, this species can utilize ‘cross-infestation’ pathways in *A. diaperinus* rearing units. However, *A. diaperinus* and the khapra beetle, *Trogoderma granarium* Everts (Coleoptera: Dermestidae), were not able to develop on 100% *T. molitor* insect meal (Rumbos et al. 2020), indicating that these species do not feed on dead insects in general, unless there are specific nutrient requirements.

*T. castaneum* was able to develop easily in all treatments tested, providing results that were comparable with those for the development of *A. diaperinus*, but, in this case, the differences between the diet that contained 100% wheat bran and the other treatments were expressed more vigorously, as indicated by the instantaneous rate of increase. Earlier studies have shown that *T. castaneum* can be also a predator on other stored product insect species, and that this characteristic may cause a spatio-temporal segregation within storage facilities from species that avoid co-occurrence with adults and larvae of *T. castaneum* (Suresh et al. 2001, Nansen et al. 2009). In the same context, *T. molitor* successfully developed on pure *A. diaperinus* meal and *A. diaperinus* meal-based substrates. This characteristic should be taken into account in units that rear both species, where ‘reciprocal’ cross infestations may be recorded. Since all three Tenebrionidae that were tested here have similar food preferences in amylaceous commodities, we estimate that there is a realistic infestation risk that has to be evaluated more thoroughly.

While we are unaware of the possible source of infestation, we hypothesize that care should be taken in the raw materials, as these may contain various stored product insects, that can subsequently expand to the standard insect rearings. From the three species tested here, *T. castaneum* has been found to infest more than 477 different types of commodities (Vaivanijkul 1973, Hagstrum et al. 2013, Hashem et al. 2014, Mailafiya et al. 2014, Ogedegbe and Edoreh 2014, Cecile et al. 2015), constituting this species as one of the ‘usual suspects’ to be introduced within the facility through raw materials, such as bran. In fact, the development of *T. castaneum* is benefited in processed amylaceous commodities and cracked grain kernels (as in the case of bran), in comparison with whole grain kernels (Hagstrum et al. 2013, Campbell et al. 2022). Even if the final product is processed with treatments that will partially control the presence of live insects, such as thermal treatment or storage in low temperatures, we suggest that the key element in reducing these losses should be the treatment of the raw materials. For instance, before its utilization in the insect rearing units, bran should be treated with insecticides in order to minimize infestations, such as the fumigant phosphine. However, phosphine may not cause complete (100%) mortality in insects eggs (Nayak et al. 2020), and thus, insect presence may be undetected in its initial post-fumigation stages. Similarly, if storage at low temperatures is to be selected for this purpose, the egg stage is likely to survive (Andreadis and Athanassiou 2017). Monitoring, not only in the raw materials but also throughout the entire insect production line could provide valuable indications for the early detection of an infestation, and certain control measures before the infestation should be expanded further within the facility. Trapping is a viable solution to this implication, for both stored product beetles and moths (Toews and Nansen 2012, Gerken and Campbell 2022), but there are still some species for which traps and attractants are not effective and their presence can be undetected, such as *T. granarium* (Athanassiou et al. 2019).

The present work provides data that show that *A. diaperinus* meals can be easily infested by *A. diaperinus* itself, but also by *T. castaneum* and *T. molitor*, that can easily build high population densities in a very short period of time. The actual impact of such a damage in the final product is poorly understood, and should be examined more thoroughly, chiefly in terms of certain quality characteristics. The increase in production of insect-based products for food and feed necessitates the need for improved storage, under the same principles that storage happens in other durable commodities, like wheat, rice, and maize. Apart from minimizing insect infestations, improved storage technologies can be adopted to be used for insect-based commodities, in conjunction with improved food security attributes.
